# Understanding Hypertension: A Metabolomic Perspective

**DOI:** 10.3390/biology14040403

**Published:** 2025-04-11

**Authors:** Inês C. R. Graça, Cláudia Martins, Fernando Ribeiro, Alexandra Nunes

**Affiliations:** 1 CICECO—Aveiro Institute of Materials, Department of Chemistry, University of Aveiro, 3810-193 Aveiro, Portugal; inescrgraca@ua.pt; 2 Institute of Biomedicine (iBiMED), Department of Medical Sciences (DCM), University of Aveiro, 3810-193 Aveiro, Portugal; claudia00@ua.pt; 3 Institute of Biomedicine (iBiMED), School of Health Sciences (ESSUA), University of Aveiro, 3810-193 Aveiro, Portugal; fernando.ribeiro@ua.pt

**Keywords:** hypertension, metabolomics, biomarkers, plasma

## Abstract

Metabolomics approaches, such as Fourier transform infrared (FTIR) spectroscopy, nuclear magnetic resonance (NMR) spectroscopy, and mass spectrometry (MS), have emerged as powerful tools for studying cardiovascular diseases (CVD), including hypertension. The use of biological fluids, like plasma and serum, has garnered significant interest due to their accessibility and potential in elucidating disease mechanisms. This review aims to summarize the current literature on the application of metabolomics techniques (FTIR, NMR, and MS) in the study of hypertension, focusing on their contributions to understanding disease pathophysiology, biomarker discovery, and therapeutic advancements. A comprehensive analysis of metabolomic studies was performed, with a particular emphasis on the diversity of altered metabolites associated with systolic blood pressure (SBP), diastolic blood pressure (DBP), and sex-related differences. Metabolomics techniques, including FTIR, NMR, and MS, provide comprehensive insights into the biochemical alterations underlying hypertension, such as amino acid and fatty acid metabolism impairment or inflammation and oxidative stress processes. This review underscores their role in advancing biomarker identification, deepening our understanding of disease mechanisms, and supporting the development of targeted therapeutic strategies. The integration of these tools highlights their potential in personalized medicine and their capacity to improve clinical outcomes.

## Simple Summary

High blood pressure, or hypertension, is a major health risk that can lead to serious conditions like heart disease and stroke. Despite medical advances, many cases remain undiagnosed or poorly managed. This review explores how the metabolomic scientific approach can help us understand hypertension pathophysiology. By analyzing biological samples such as plasma and serum, metabolomics techniques like nuclear magnetic resonance (NMR) and mass spectrometry (MS) reveal changes in metabolism linked to high blood pressure. Key findings highlight disruptions in amino acids, fatty acids, oxidative stress, and inflammation, which contribute to the disease. Identifying these metabolic markers can lead to earlier diagnosis and more personalized treatments, potentially improving health outcomes for millions of people worldwide.

## 1. Introduction

Hypertension is a global, multifactorial condition that represents one of the most important preventable causes of death worldwide [1,2,3]. Hypertension is defined as the level of blood pressure (BP) at which the benefits of treatment (pharmacological or non-pharmacological) clearly exceed the risks of treatment, as documented by clinical trials. In Europe, it is defined as systolic blood pressure (SBP) ≥ 140 mmHg and/or diastolic blood pressure (DBP) ≥ 90 mmHg [4,5].

Despite the advances in medical therapy in recent decades, hypertension remains a major risk factor for cardiovascular diseases (CVDs), such as stroke, heart failure, and acute myocardial infarction, as well as chronic kidney disease and even dementia. This disease is commonly asymptomatic and almost one-half of individuals are not aware that they have hypertension, and in those diagnosed, hypertension is not adequately treated in approximately one-half of them [6]. Over the next few years, it is expected that the incidence of hypertension will increase by 60%, which will have a direct impact on the costs of the health system [7].

Although hypertension causes are still not entirely clear, there are several factors associated with this increase in hypertension prevalence, such as population growth and aging, usually influenced by low levels of physical activity, unhealthy diets, alcohol and tobacco consumption, and exposure to stress [3,7]. Typically, hypertension is associated with dyslipidemia, inflammation, and oxidative stress [8].

During the development of hypertension, the pathogenesis and pathophysiology of the disease may change, even before significant increases in clinic upper arm BP are recognized. Establishing a new ‘omics’ approach is essential for early prediction and better elucidation of the molecular mechanisms of hypertension [9,10]. This could support prevention, management, new therapeutic strategies, and improved diagnostic methods [11].

Metabolite profiling has generated growing interest in the field of hypertension since the initial discovery of a correlation between serum metabolite profiles and BP in patients with hypertension [3]. Over time, numerous efforts have been made to understand the connection between genetic and metabolic characteristics, as well as the reduction in BP [3].

Therefore, this paper aims to provide a comprehensive and updated review of different metabolomic approaches for identifying changes in plasma or serum metabolic profile in individuals with hypertension. By examining key metabolic pathways, it contributes to a deeper understanding of hypertension pathogenesis.

## 2. Metabolomics and Hypertension

Metabolomics is an ‘omics’ approach that, by using analytical techniques with the combination of cheminformatics, bioinformatics, and statistical analyses, studies the global metabolic profile and unique chemical fingerprint of small molecules or metabolites in biological samples, such as cells, tissues, and body fluids, that are related to various cellular metabolic processes [11,12].

Metabolites such as peptides, lipids, fatty acids, or amino acids can vary profoundly in polarity, size, and concentration, from hydrophilic, polar metabolites with low molecular weight, to hydrophobic, non-polar high-molecular-weight metabolites, providing extensive information on the physiological state and metabolic pathways [10,13,14]. The perturbations of the metabolic profile due to various environmental or physicochemical conditions offer new insights into disease pathogenesis and treatment approaches [14,15].

Due to the diversity of metabolites, it is extremely technically challenging to perform the unbiased detection, identification, and quantification of the entire metabolome [16]. Indeed, there is not yet a single analytical approach that can either detect or quantify all metabolites present in human samples. However, there have been employed multiple analytical techniques complementing each other in the coverage of several metabolites with some success [10].

Two different metabolomic strategies can be applied to analyze metabolites: targeted and untargeted metabolomic approaches. The first, targeted metabolomic approach, is based on a hypothesis-driven manner and used for biomarkers validation [17,18]. It is the measurement of a selected group of metabolites, normally focusing on one or more related pathways [19]. Usually, it is performed using mass spectrometry (MS) [11]. In contrast, untargeted approaches allow the unbiased detection of the full set of metabolites in biological samples, offering the opportunity for the discovery of novel metabolites and metabolic pathways without prior knowledge of the identified metabolites [20]. Untargeted metabolomics can be conventionally performed using nuclear magnetic resonance (NMR) and MS approaches to measure and quantify as many metabolites as possible [21]. Currently, several other methods are used in metabolomics, such as NMR-coupled MS, liquid chromatography coupled to MS (LC–MS), gas chromatography coupled to MS (GC–MS), and Fourier transform infrared (FTIR) spectroscopy [13,14]. Then, multivariate statistical analysis and pattern recognition tools, such as principal component analysis (PCA) and partial least square discriminant analysis (PLS-DA), are used to compare and identify features that allow for the distinction between complex metabolic profiles [14].

NMR spectroscopy is a quantitative method that offers precise structural information that allows relatively easy metabolite identification [22]. It is also not destructive and does not require extensive sample preparation [23]. However, it has relatively low analytical sensitivity, limiting detection to high-abundance metabolites [22,23]. Furthermore, typical one-dimensional NMR often results in complex metabolite profiles where low-abundance metabolites may be masked by high-abundance ones with a similar chemical shift. This challenge can be addressed with sophisticated and expensive techniques, such as two-dimensional NMR, or by simplifying biological matrices before analysis [10]. On the other hand, MS coupled with separation techniques such as LC or GC presents a higher sensitivity and can detect numerous very-low-abundance metabolites [24]. However, unambiguous assignments and identification of signals remain difficult and time-consuming, representing a significant bioinformatics (and experimental) challenge that can only be partially resolved [25]. Besides that, MS has low reproducibility compared with NMR, due to variations in retention times and signal intensity between samples and instruments [26]. Nevertheless, MS-based metabolomics is still the preferred choice for detecting low-abundance metabolites or resolving large numbers of metabolites simultaneously [26,27]. More recently, there has been a significant development in FTIR spectroscopy in metabolomics, as it has several useful advantages to studying biological samples, producing highly sensitive and reproducible results, and allowing the screening of a biological sample within a few minutes [28,29]. Nevertheless, compared to MS or NMR-based approaches, it does not allow the identification of specific molecules but allows the identification of a specific spectral signature [28]. Despite all FTIR advantages, the predominant techniques employed on hypertension are still MS and NMR.

**Figure 1:**
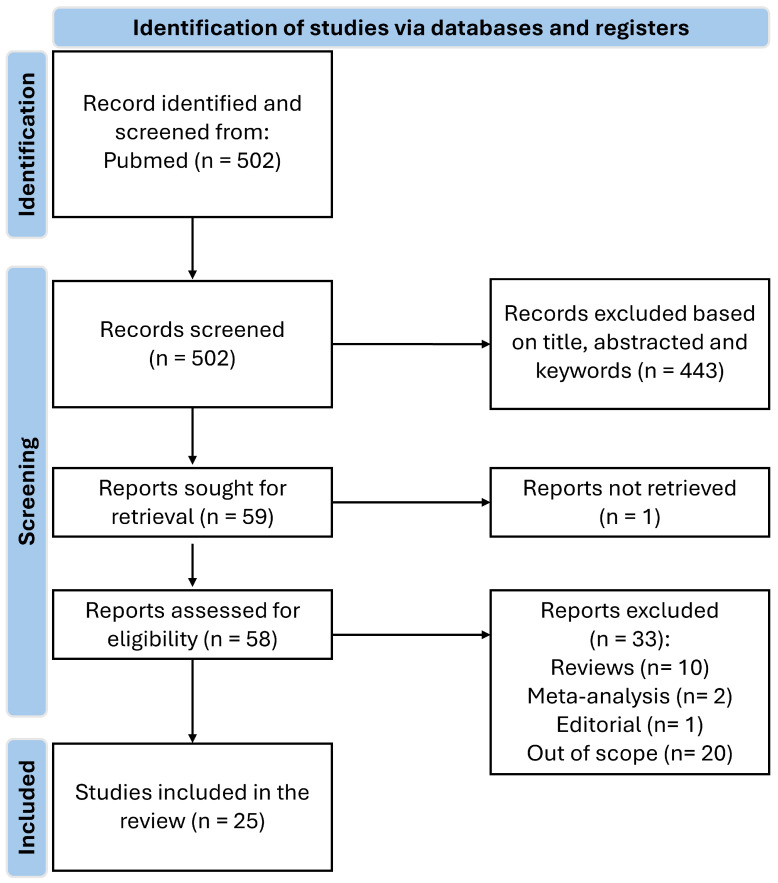
Flowchart of the search conducted in PubMed resulting in the research articles included in this review and presented in Table 1.

Current research on hypertension has been focused on the effects of lifestyle and environmental factors, disease processes, and drug response, which could improve the understanding of the metabolic disorders of this disease [14]. In recent decades, many metabolomics studies on hypertension have been conducted, including human and animal studies [30]. Figure 1 illustrates the review process. In brief, out of 502 studies, 58 were eligible for full-text analysis, of which 33 were excluded and 25 were included in the review.

Relevant studies were identified by searching the PubMed database. The search terms were the following: “((hypertension[Title/Abstract]) NOT (resistant[Title/Abstract]) NOT (pulmonary[Title/Abstract]) NOT ((preeclampsia[Title/Abstract]) OR (pregnant[Title/Abstract]) OR (pregnancy[Title/Abstract]))) NOT (heart failure [Title/Abstract]) AND ((metabolomics[Title/Abstract]) OR (metabolome[Title/Abstract]) OR (metabolite[Title/Abstract])) AND (human) NOT (urine OR urinary) AND (English[Language])” and “((hypertension[Title/Abstract]) AND (FTIR[Title/Abstract] OR FT-IR[Title/Abstract] OR vibrational spectroscopy[Title/Abstract])) AND (human NOT (mice OR rat)) AND (English[Language])”. The search was limited to studies published between January 2015 and 29 January 2025. The inclusion criteria were: (i) articles written in English; (ii) observational or experimental studies; (iii) studies assessing metabolites related to hypertension or SBP and DBP. Articles referring to other types of hypertension or related diseases or articles whose samples used were urine, were excluded. Studies not conducted in humans, such as animal or in vitro studies, review articles, meta-analysis, qualitative studies, news, magazines, research protocols, reports, thesis, dissertations, abstracts, unpublished work, books, book chapters, guidelines, position papers, and studies that were not within the scope of our study were excluded. In Table 1, it is presented a summary of the metabolomic studies included in this review.

Through our search, we also identified a few reviews with a similar design. However, the novelty of our article lies in its comprehensive compilation of more recent studies, published between January 2015 and January 2025. Notably, over half of all articles in this field were published during this ten-year period, highlighting the continuous advancements in metabolomics as a tool for studying hypertension.

These studies have suggested several potential metabolic pathways responsible for hypertension, such as inflammation, oxidative stress, lipid profile, and gut microflora (Table 1) [10,31,32,33]. Curiously, these pathways can also create a cascade of events that may result in a complication in the severity of the condition [14].

**Table 1:** Summary of the studies on hypertension metabolism, indicating the type of sample used, the techniques, the metabolites identified in each study, and the main findings of each study.

Sample Type	Techniques	Metabolites	Main Findings	Reference
Plasma	LC–MS/MS	↑ Indole-3-carboxaldehyde; 4-aminohippuric acid; L-glutamic acid; saccharopine; DL-glutamate; L-kynurenine; L-valine; L-isoleucine; L-tyrosine; benzaldehyde; L-(-)-fucose; glyceraldehyde; pseudouridine; trans-aconitic acid; threonic acid; cis-aconitic acid; N-acetyl-L-alanine; 4-acetylbutyrate; citraconic acid; 2,4-undecadiene-8,10-diynoic acid isobutylamide; sphingomyelin (d18:1/18:1(9Z)); millefin; cis, cis-muconic acid; 2,4′,5,7-tetrhydroxyflavanone; L-cystine; acetylhomoserine; (3S,5S))—3,5-diaminohexanoate; N-acetylserine; pyrrolidonecarboxylic acid; 2-ocovaleric acid; 3,3,5-triiodo-L-thyronine-beta-D-glucuronoside; L-ribulose; L-glucose; (2R)-2-hydroxy-2-phenylethyl; 2-furoic acid; isosalsolidine; 3-propylidene-1(3H)-isobenzofuranone; piperidine; 2,4′,5,7-tetrahydroxyflavanone; N’-hydroxysaxitoxin; 3-(3,4-dihydroxyphenyl)prop-2-enoic acid; coumaric acid; bergapten; phenylacetaldehyde; 2-phenylacetamide; romucosine A; alpha-D-glucose; prostaglandin D2 ↓ (9S, 10E, 12Z, 15Z)-9-hydroxy-10,12,15-octadecatrienoic; falcarinolone; dolichosterone; deoxyribose 5-phosphate	L-cystine, L-glutamate acid, DL-glutamate, cis-aconitic acid, citraconic acid, and 2-furoic acid levels were positively associated with both SBP and DBP.	Chen et al. (2024) [34]
Plasma	LC–MS/MS	↑ Uric acid; leucine ↓ LysoPC (18:2)	Sexual dimorphism of the metabolome may contribute to sex differences in hypertension. Higher levels of uric acid and leucine and lower levels of a LysoPC were associated with hypertension.	Couch et al. (2024) [35]
Serum	LC–MS/MS	↑ Fatty acid 20:2; palmitoylcarnitine; palmitoleoylcarnitine; fatty acid 18; N-acetyleucine; PE (18:0_18:2); hydrocortisone; triglycerides (22:6_34:0); PC (14:0_20:5); phe-phe ↓ PC (15:0_18:2)	Results revealed the metabolic connectivity and specificity across multiple chronic metabolic diseases in terms of different metabolite classes.	Zhang et al. (2024) [36]
Serum	UPLC–MS	↑ C2; butyric acid; ethanolamine ↓ Pyruvate	Hypertension plasma patients exhibited significantly higher levels of creatinine and LDL-C.	López-Romero et al. (2024) [37]
Plasma	high-throughput NMR	Related to SBP: ↑ ApoB; LDL cholesterol; total triglycerides ↓ ApoA1; HDL cholesterol Related to DBP: ↑ ApoA1; total triglycerides ↓ ApoB; HDL cholesterol; LDL cholesterol	HDL cholesterol and low-density lipoprotein cholesterol were found to be potential causal factors for pulse pressure (PP) elevation. Sub-lipoproteins, genetically predicted atherogenic lipoprotein particles had the most likely causal impact on increasing PP.	Liu et al. (2024) [38]
Plasma	UPLC–MS/MS coupled with a heated electrospray ionization (HESI-II) source	↑ {stearidonate [18:4n3], hexadecadienoate [16:2n6]; 9 and 13-S-hydroxyoctadecadienoic acid (HODE); linolenate [alpha or gamma; (18:3n3 or 6)]}; N6-carbamoylthreonyladenosine; 2,3-dihydroxy-5-methylthio-4- pentenoate (DMTPA)	Six metabolites, including lipids, an amino acid and a nucleotide, were found to be associated with the increased risk of hypertension.	Al Ashmar et al. (2024) [39]
Plasma	flow-injection tandem MS	↑ Alanine; valine; leucine; phenylalanine; proline; C2; C3; C3DC; C4; C4DC; C4OH; C5; C5OH; C5DC; C6; C8; C8:1; C10; C10:1; C12; C14; C14:1; C14:2; C14OH; C16; C16OH; C16:1OH; C16:1; C18; C18:1; C18OH; C18:1OH; C18:2OH ↓ Aspartic acid; glycine; serine	Five metabolites were identified as predictors of stage 2 hypertension: C0; C12; C14:1; C14:1 and glycine.	Arjmand et al. (2023) [40]
Plasma	UPLC–MS/MS	Related to SBP in women: ↑ dihomo-lineoylcarnitine; 4-hydroxyphenylacetateglutamine; vanillactate; DMTPA; 1-methyladenosine Related to SBP in Men: ↑ sphingomyelins; N-formylmethionine; conjugated bile acids; N-acyl amino acids	Plasma metabolite associated with BP in a sex-specific way: catecholamine derivatives are predictors for BP in women, while sphingomyelins were more important in men.	Verhaar et al. (2023) [41]
Plasma	LC–MS/MS	ES-: ↑ methadone-d9; N-dodecanoylshinganine; DL-arginine ↓ 4-methylcarbostyril; (E)-4-methoxycinnamic acid; guaietolin; (E)-3,4,5-trimethoxucinnamic acid; 3-hydroxy-3-(2,3,4-trimethoxyphenyl)propanic acid; 1-(1Z-hexadecenyl)-sn-glycero-3-phosphocholine; epi-jasmonic acid; (+-)-(2E)-abscisic acid; 2-amino-3-hydroxyoctadecyl dihydrogen phosphate; sphingosine 1-phosphate; umbelliferone; LU3453000; DL-lactic acid; hexitol; furaneol; sinaticin; 2-deoxyhexopyranose; chlorphentermine; [(4Z)-5-(methylsulfanyl)-4-penten-2-yn-1-yl]benzene; 1-O-arsonopentofuranose; 2,4-dichlorotoluene; S-3-oxodecanoyl cysteamine; lauramide; (2S)-2-amino-8-hydroxyoctanoic acid; crotonic acid; guvacine; glycine-leucine; 4-oxoproline ES+: ↑ 4-hydroxycyclophosphamine; methyl isoquinoline-3-carboxylate; benzothiazole; N-dodecanoylshinganine; DL-arginine ↓ 1-O-arsonopentofuranose; 2,4-dichlorotoluene; (2S)-2-amino-4-hexynoic acid; NLK; sinaticin; maltitol; 3,4,5-trihydroxy-6-(h)-oxan-2-yl-3-hydroxy-4,5-dimethoxybenzoate; [(4Z)-5-(methylsulfanyl)-4-penten-2-yn-1-yl]benzene; chlorphentermine; 6a,7,8,12-tetrahydroxy-3-methyl-(1,7),6a,12,12a-decahydrotetraphene-1,12-dione; (2Z)-3-{5-[(4Z)-5-(methylsulfanyl)-4-penten-2-yn-1-yl]-2-furyl acrylaldehyde; D-(+)-maltose; 3-methylglutarate; 5-acetylgidydro-2-(3H)-furanone; 2-deoxyhexopyranose; hexitol; S-3-oxodecanoyl cysteamine; lauramide; 4-hydroxycoumarin; 2-amino-3-hydroxyoctadecyl dihydrogen phosphate; shingosine 1-phosphate; hydroxytriazolam; 4-coumaric acid; guaietolin; 3-(6,7-dimethoxy-1,3-benzodioxol-5-yl)-2-propen-1-ol; P-Gal; 4-methylcarbostyril; epi-iasmonic acid; (+-)-(2E)-abscisic acid; 3-phenylpropanic acid; 1-[(1Z,9Z)-octadecadienyl]-sn-glycero-3-phosphocholine; 1-[(11Z)-octadecenoyl]-sn-glycero-3-phosphocholine; 1-(1Z-hexadecenyl)-sn-glycero-3-phosphocholine; 2-aminoethyl(2R)-3-[(1Z)-1-hexadecen-1-yloxy]-2-hydroxypropyl hydrogen phosphate	Correlations between plasma metabolites and microbiota suggested impairment of interactions between metabolites and microbes in patients with hypertension.	Chen et al. (2023) [42]
Plasma (from Chen et al. [43])	UPLC–MS/MS (from Chen et al. [43])	Related to DBP: ↑ N-alpha-acetylornithine; N-acetyl-2-aminoadipate; N-acetylarginine; N-acetylglutamine; N6-acetyllysine; N-acetylcitrulline; behenoyl dihydrosphingomyelin [d18:0/22:0] ↓ 2-hydroxyoctanoate; N2,N2-dimethylguanosine; alliin; N-delta-acetylornithine; 1-(1-enyl-stearoyl)-2-arachidonoyl-GPE (P-18:0/20:4) Related to SBP: ↑ N1-methyl-2-pyridone-5-carboxamide; X-12847; X-12822; ferulic acid 4-sulfate; X-12839; X-11381; N-formylmethionine; X-15486 ↓ X-25420; Imidazole propionate; 5-hydroxy-2-methylpyridine sulfate; 3-hydroxy-2-methylpyridine sulfate; gamma-glutamylthreonine; mannonate; 1-methyl-4-imidazoleacetate; X-25419; CMPF; X-24531; X-21364; N-succinyl-phenylalanine; hydroxy-CMPF; phenylacetylglutamate	12 metabolites had a causal effect on DBP, and 22 metabolites had a causal effect on SBP.	Dai et al. (2023) [44]
Plasma and Serum	MWAS/MS or NMR or other multianalyte analytical technology	↑ Cortisol; cortisone; glycocholic acid; chenodeoxycholic acid glycine conjugate; 5-androstenediol; L-acetylcarnitine; tetradecanedioic acid; steraridonic acid; LysoPE (16:0/0:0); LysoPE (20:4(5Z,8Z,11Z,14Z)/(0:0); glycerylphosphorylethanolamine; homocitrulline; L-palmitoylcarnitine; L-isoleucine; L-leucine; N-acetyl-L-alanine; L-lactic acid; L-malic acid; Ketoleucine; 3-methyl-2-oxovaleric acid; myo-inositol; pantothenic acid; glycerol; erythritol; indolelactic acid; theophylline; caffeine; pyridoxine ↓ Glycine; hydrocinnamic acid; L-histidine; L-serine	Using a multi-ethnic cohort reported 5 novel hypertension-associated metabolites and confirmed 27 previous hypertension associations. Of these, 32 metabolite associations, predominantly lipid (steroids and fatty acyls) and organic acids (amino-, hydroxy-, and keto-acids) remained after further adjusting for comorbidities and dietary intake.	Louca et al. (2022) [45]
Serum	NMR	↑ Acetoacetate; alanine; albumin; ApoB/Apo 1; ApoA1; ApoB; β-hydroxybutyrate; esterified-C; glucose; glycerol; glycoprotein acetyls; HDL cholesterol; HDL2 cholesterol; HDL3 cholesterol; isoleucine; LDL cholesterol; lactate; leucine; monounsaturated fatty acids; phenylalanine; PCs; phosphoglycerides; pyruvate; remnant-C; saturated fatty acids; sphingomyelins; triglycerides/phosphoglycerides; total cholesterol; total cholines; total fatty acids; total free cholesterol; total triglycerides; triglycerides in HDL; triglycerides in LDL; triglycerides in VLDL; VLDL cholesterol; VLDL size ↓ Acetate; unsaturation degree; glutamine; glycine; HDL size; linoleic acid%; LDL size; omega-3%; omega-6%; polyunsaturated fatty acids%	Among other findings, LDL-derived and VLDL-derived cholesterol and glucose metabolism abnormalities are associated with hypertension patients.	Palmu et al. (2022) [46]
Plasma	UPLC–MS/MS	↓ Acetate; isobutyrate; butyrate; isovalerate	Microbiota and bacterial metabolites were used to classify non-treated hypertension in first-degree phase patients.	Calderón-Pérez et al. (2020) [47]
Plasma	LC–MS and GC–MS	Related to DBP: ↑ Ceramide (C18:1,C24:0); triacylglycerol (C16:0, C16:1); oleic acid (C18:cis [9]1); total glycerolipids ↓ Cholesterylester C16:0	Ceramide, triacylglycerol, total glycerolipids, and oleic acid were positively associated with longitudinal diastolic BP change. Cholesterylester levels were inversely associated with longitudinal diastolic BP change.	Lin et al. (2020) [32]
Plasma	LC–MS	Related to Men: ↑ C4 acylcarnitine; LysoPC a C26:1 ↓ Ornithine; tryptophan; leucine; valine; histidine; threonine; methionine; lysine; spermidine Related to Women: ↑ Arginine; citrulline; tryptophan; histidine; tyrosine; phenylalanine; leucine; isoleucine; glutamine; methionine; lysine; kynurenine; taurine; alpha-AAA; C10, C12 and C12:1 acylcarnitines ↓ Spermine Both: ↑ Acetyl-ornithine; PC; LysoPC a C28:1; sphingomyelins; hydroxyproline; SDMA/total DMA ↓ LysoPC C16:0	Both sexes showed a considerable increase in PCs, a decrease in C16:0 with an increase in C28:1 LysoPCs, an increase in sphingomyelins, as well as an increase in symmetric dimethylarginine (SDMA), acetyl-ornithine, and hydroxyproline. Twenty-nine metabolites, involved in phospholipidic and cardiac remodeling, arginine/nitric oxide pathway, and antihypertensive and insulin resistance mechanisms, discriminated the metabolic sexual dimorphism of hypertension.	Goïta et al. (2020) [48]
Serum	UPLC–MS/MS	↑ Formiminoglutamate; 1-palmitoyl-2-linoleyl-GPE (16:0/18:2); 1-stearoyl-2-arachidonoyl-GPE (18:0/20:4); 1-stearoyl-2-linoleoyl-GPE (18:0/18:2); 1-stearoyl-2-oleoly-GPE (18:0/18:1); *N*-palmitoyl-sphinganine (d18:0/16:0); *N*-stearoyl-sphinganine (d18:0/18:0); fibrinopeptide B (1-13); gamma-glutamylisoleucine; ethyl glucuronide; X-24337; urate; glucose ↓ Oxalate; threonate; thromboxane B2; 5-methylutidine (ribothymide); fibrinopeptide B (1-11); fibrinopeptide B (1-12); tartronate (hydroxymalonate); X-17367; X-21752; serine	24 novel metabolites were identified: 3 amino acid and nucleotide metabolites; 7 cofactor and vitamin or xenobiotic metabolites; bacterial/fungal, chemical, and food component sub-pathways; 10 lipid metabolites from the eicosanoid, PC, PE, and sphingolipid metabolism sub-pathways.	He et al. (2020) [49]
Platelet	ATR-FTIR and Raman Spectroscopy	↓ cholesterol band area (2930 cm^−1^) Shift in: Phosphatidylinositol (589 cm^−1^) PE (760 cm^−1^) PC (720 cm^−1^) Phosphatidylserine (595 and 785 cm^−1^)	Significant modifications in the major lipid composition and cholesterol content of the plasma membrane in hypertension platelets were shown.	García-Rubio et al. (2019) [50]
Serum	UPLC/MS and GC/MS	↑ Hexadecanoic acid; glycerol; hexadecenoic acid; Tetradecanoic acid; LysoPC (16:1); PC (14:0/18:1); PC (16:0/22:3); PC (18:0/20:4); DG (15:0/18:3); DG (15:0/18:4/0:0); DG (14:0/22:5); L-acetylcarnitine; cis-5-tetradecenoylcarnitine; C12; trans-hexadec-2-enoyl carnitine; 4,8 dimethylnonanoyl carnitine; palmitic amide; N-Acetylarylamide; 3-oxododecanoic acid; oleic acid; (±)-10-HDoHE; 4-hydroxybenzaldehyde; 3-hydroxyhippuric acid; hexadecanedioic acid	26 metabolites were identified and are mainly involved in fatty acid metabolism, glycerophospholipid metabolism, alanine, aspartate, and glutamate metabolism, and are implicated in insulin resistance, vascular remodeling, macrophage activation, and oxidized LDL formation.	Ke et al. (2018) [51]
Blood (Dried Blood Spot)	ESI–MS	↑ C4/C8; C5OH/C8; C3DC/C10; C10:2/C10; ornithine/citrulline; ornithine; C5DC/C8 ↓ Glycine; C8; C10; C12; glycine/alanine; phenylalanine/tyrosine; C14:1; C14:1/C16; C5DC; C10:1; C14OH; C14:2; C4DC	Glycine, ornithine, decanylcarnitine and the ratios Ornithine/citrulline; and phenylalanine/tyrosine and -hydroxyisovalerylcarnitine/octanoylcarnitine are characteristic of patients with hypertension.	Bai et al. (2018) [52]
Serum	NMR	↑ Alanine; adenine; methionine; pyruvate; uracil ↓ Arginine	Alanine, arginine, methionine, pyruvate, adenine, and uracil showed excellent correlation in both isolated elevated DBP cases and combined elevated systolic–diastolic blood pressure cases.	Ameta et al. (2017) [53]
Serum	AbsolutelDQ p150 Kits based on flow injection analysis tandem MS	Lower risk of developing hypertension: ↑ serine; acyl-alkyl-PCs C42:4 and C44; glycine Higher risk of developing hypertension: ↑ diacyl-PCs C38:4 and C38:3	Higher concentrations of serine, glycine, and acyl-alkyl-PCs C42:4 and C44:3 tended to be associated with higher predicted 10-year hypertension-free survival and diacyl-PCs C38:4 and C38:3 with lower predicted 10-year hypertension-free survival.	Dietrich et al. (2016) [8]
Serum	GC/MS	↑ Talose; lyxose; methylmalonic acid; malonic acid; shikimic acid; glucose-1-phosphate ↓ Threonine; nicotinoyl glycine; glycine; phenylalanine; S-carboxylmethycysteine; tyrosine; Aspartic acid; glycine-proline; galactose; methyl-β-D-galactopyranoside; dihydroxyacetone; melezitose; oxalic acid; thymol; noradrenaline; 2-aminophenol; 2-methoxyestrone; alpha-tocopherol; octadecanol; 2-aminoethanethiol	Threonine and phenylalanine were negatively associated with the risk of future hypertension. A higher level of lyxose was associated with a higher risk of hypertension.	Hao et al. (2016) [54]
Plasma	MRM–MS	↑ Oleic acid ↓ Myo-inositol	The results showed that oleic acid (OA) and myoinositol (MI) were the most important differential metabolites between the hypertensive plasma and the healthy plasma.	Yang et al. (2016) [15]
Serum and Plasma	MRM–MS	Related to DBP: ↓ Lactate; C8; hexadecanedioate; tetradecanedioate; 10-heptadecenoate (17:1n7); 5-dodecenoate (12:1n7); cortisol; caffeine Related to SBP: ↑ Lactate; C8; hexadecanedioate; tetradecanedioate; 10-heptadecenoate (17:1n7); 5-dodecenoate (12:1n7); cortisol; caffeine Both: ↑ Dihomo-linoleate (20:2n6); palmitate (16:0); 4-androsten-3β,17β-diol disulfate 1 ↓ Phenylacetylglutamine; stearoylcarnitine; nonadecanoate; HWESASXX	Hexadecanedioate showed a concordant direction of effect for both BP and mortality, while in contrast, the direct association between dihomo-linoleate(20:2n6) or caffeine and BP did not translate into increased mortality risk.	Menni et al. (2015) [55]
Plasma	GC–MS	↑ oxalic acid; fumaric acid; glycerol; adenine; pyrophosphate; uric acid ↓ L-valine; L-isoleucine; glycine; L-threonine; L-methionine; ornithine; L-asparagine; L-glutamine; citrulline; L-lysine; L-tyrosine; L-tryptophan; L-cystine; capric acid	Disorders of amino acid metabolism might play an important role in predisposing young men to developing hypertension.	Wang et al. (2015) [56]

↑, increased levels; ↓, decreased levels. Abbreviations: AAA, aromatic amino acids; ApoA1, alipoprotein A1; ApoB, alipoprotein B; ATR, attenuated total reflectance, CMPF, 3-carboxy-4-methyl-5-propyl-2-furanpropanoate; C2, acetylcarnitine; C3, propionylcarnitine; C3DC, malonylcarnitine; C4, butyrylcarnitine; C4DC, methylmalonyl-/succinylcarnitine; C4OH, 3-OH-iso-/butyrylcarnitine; C5, isovalerylcarnitine; C5OH, 3-OH-isovalerylcarnitine; C5DC, glutarylcarnitine; C6, hexanoylcarnitine; C8, octanoylcarnitine; C8:1, octenoylcarnitine; C10, decanoylcarnitine; C10:1, decenoylcarnitine; C10:2, decadienoylcarnitine; C12, dodecanoylcarnitine; C14, tetradecanoylcarnitine; C14:1, tetradecenoylcarnitine; C14:2, tetradecadienoylcarnitine; C14OH, 3-OH-tetradecanoylcarnitine; C16, hexadecanoylcarnitine; C16OH, 3-OH-hexadecanoylcarnitine; C16:1OH, 3-OH-hexadecenoylcarnitine; C16:1, hexadecenoylcarnitine; C18, octadecanoylcarnitine; C18:1, octadecenoylcarnitine; C18OH, 3-OH-octadecanoylcarnitine; C18:1OH, 3-OH-octadecenoylcarnitine; C18:2OH, 3-OH-octadecadienoylcarnitine; DBP, diastolic blood pressure; DG, diglyceride; DMA, dimethylarginine; DMTPA, 2,3-dihydroxy-5-methylthio-4-pentenoic acid; ESI, electrospray ionization; FTIR, Fourier transform infrared spectroscopy; GC, gas chromatography; HDL, high-density lipoprotein; LC, liquid chromatography; LDL, low-density lipoprotein; MRM, multiple reaction monitoring; MS, mass spectrometry; MWAS, microbiome-wide association study; NMR, nuclear magnetic resonance; PC, phosphatidylcholine; PE, phosphatidylethanolamine; PP, pulse pressure; SDMA, symmetric dimethylarginine; SBP, systolic blood pressure; UPLC, ultrahigh performance liquid chromatography; VLDL, very-low-density lipoprotein.

Table 1 provides a summary of various studies that have identified a range of metabolites from diverse metabolic pathways associated with hypertension or alterations in SBP and DBP. These articles mainly use MS-based techniques, with some using NMR spectroscopy and finally, one single study using vibrational spectroscopy techniques, namely ATR-FTIR and Raman spectroscopy. Despite the lack of studies, it should be noted that there has been a significant development in vibrational spectroscopy to study metabolome associated with physiological changes in biological fluids. FTIR spectroscopy has been demonstrating significant potential in the early and minimally invasive diagnosis of several pathologies, including cancer [29] and dementia [57]. Compared to NMR and MS-based approaches, it does not identify specific molecules but allows screening of a biological sample within minutes and detection of a specific spectral signature [28].

Table 1 highlights several metabolites frequently reported in these studies, namely alanine, leucine, isoleucine, valine, glycine, lactate, serine, arginine, methionine, glycerol and acylcarnitines. These findings indicate that hypertension is associated with impairments in multiple metabolic pathways, such as amino acid metabolism [8,34,39,40,41,42,44,45,46,52,53,54,55,56], lipid metabolism [32,36,37,38,39,40,41,42,46,47,48,50,51,52], fatty acid metabolism [15,32,36,37,40,48,51,52,55], energy metabolism [34,37,45,46,49,53,55], cholesterol metabolism [32,38,46,50], carbohydrate metabolism [42,46,49,54], steroid hormone biosynthesis [34,45,46,49,53,55]. Besides these metabolic pathways, the metabolites presented in Table 1 are also involved in inflammation [34,35,41,42,46,48,51] and oxidative stress [35,46,48,51]. Overall, these results highlight the complex metabolic alterations that occur in individuals with hypertension.

To better understand the role of these metabolites and pathways in hypertension, we will now focus on amino acid metabolism and fatty acid metabolism, inflammation, and oxidative stress as they seem to be highly disturbed in hypertension pathogenesis, being referenced in many studies presented above.

### 2.1. Amino Acid Metabolism

As presented in Table 1, amino acid metabolism has been repeatedly found to be impaired in patients with hypertension. Amino acids, including leucine, alanine, and phenylalanine (except in [54]) were observed at higher concentrations in blood samples of patients with hypertension [34,35,40,45,46,53]. However, aspartic acid, glycine, valine (except in [34,40]), serine, isoleucine (except in [34,56]), threonine, and tyrosine (except in [34]) were found to have lower concentrations [40,45,46,49,52,54,56]. In 2020, Goïta et al. [48] observed differences in amino acids between women and men, namely in tryptophan, leucine, histidine, ornithine, citrulline, methionine, and lysine. Besides this, arginine, ornithine, and methionine were found to be increased ([42,52,53], respectively) and decreased ([53,56], respectively) in different studies. The discrepancies observed in metabolomic studies regarding certain amino acids (specifically phenylalanine, valine, isoleucine, and tyrosine) might be attributed to variations in study design. There are still other amino acids mentioned, although they were detected with less frequency within the studies presented in Table 1.

Amino acids are the foundation components for peptides and proteins, playing crucial roles in several processes like cell signaling, metabolism, gene regulation, and neurotransmission [58]. In hypertension, there is evidence that amino acids may play an important role in its pathogenesis, namely branched-chain amino acids (BCAAs; leucine, isoleucine, and valine) and aromatic amino acids (AAAs; phenylalanine, tryptophan, and tyrosine), that seem to be altered in individuals with hypertension [59].

Several hypotheses have emerged in the scientific community that relate these amino acids to hypertension [59]. One suggests that increased levels of circulating BCAAs might increase the angiotensin-converting enzyme activity, a key regulator of BP, which results in increased binding of these amino acid residues to the enzyme [60,61]. Moreover, the extended activation of the mTORC1 pathway in the vascular system due to BCAAs may also explain the association between these amino acids and BP, as it can lead to several physiological alterations, including changes in sympathetic nerve activity [62], proliferation of vascular smooth muscle cells [63], and kidney damage [64], which can subsequently contribute to the onset of hypertension [59].

Regarding AAAs, phenylalanine can influence the formation of a cofactor, tetrahydrobiopterin (BH4), that relates to the relaxation of the endothelium during the hydroxylation process of AAAs [59]. Thus, an excess of AAAs can potentially disrupt the vasoactive features of BH4, thereby negatively impacting the endothelium [65]. In addition, tryptophan plays a role in vascular function as it serves as a precursor for serotonin synthesis [66]. Animal studies have demonstrated a decrease in BP following tryptophan consumption [67]. Tryptophan-containing peptides, formed during the digestion of dietary protein, may also inhibit the renin–angiotensin axis by influencing the activity of angiotensin-converting enzyme [68].

Methionine emerges as another significant amino acid in the development of hypertension, exerting an indirect influence on BP by raising homocysteine levels. Homocysteine, a metabolic byproduct of methionine, when elevated, can compromise endothelial function by stimulating the production of asymmetrical dimethylarginine (ADMA), consequently inhibiting the synthesis of nitric oxide (NO) [69].

Finally, it is suggested that glycine, a nonessential amino acid, plays a role in maintaining normal BP through various mechanisms, such as contributing to glutathione synthesis, which helps reduce excess aldehydes and free radicals, promoting NO production, and participating in the synthesis of elastin and collagen. Thus, a deficiency in glycine results in endothelial dysfunction, reduced availability of NO, and impaired aortic elasticity, consequently leading to the development of hypertension [40].

### 2.2. Fatty Acid Metabolism

There has been evidence of a strong association between hypertension and abnormalities in lipid metabolism and dyslipidemia [14]. As shown in Table 1, several studies reported alterations in metabolites related to fatty acid metabolism such as oleic acid, ceramide, and short-chain fatty acids (SCFAs) in patients with hypertension [15,32,37,46,47,51]. In 2022, Palmu et al. [46] also showed an increase in the percentage of monounsaturated fatty acids (MUFA), percentage of saturated fatty acids, and total fatty acids and a decrease in the percentage of omega-3, omega-6, and polyunsaturated fatty acids.

Lin et al. [32] observed an increase in ceramide associated with longitudinal DBP changes. Ceramide was regarded as the central intermediate in the sphingolipid biosynthetic pathway. It mediates vascular dysfunction by inhibiting endothelial nitric oxide synthase (eNOS)-serine/threonine protein kinases heat shock protein 90 signaling complex [32]. Additionally, it also induces endothelium-dependent contraction by triggering the release of the pro-inflammatory compound thromboxane A2. This release is facilitated through the calcium-independent phospholipase A2 (iPLA2), cyclooxygenase-1 (COX-1), and thromboxane synthase (TXAS) pathways [14].

Oleic acids were suggested to have a vasodilating effect, thereby causing a reduction in BP [70,71]. A recent study also showed the protective action of oleic acid against cellular lipotoxicity, attenuating oxidative stress, endoplasmic reticulum stress, inflammation, and apoptosis [72], although it seems to be increased with hypertension [15,32,51]. A review from 2006 of studies conducted in the Mediterranean countries reported that MUFA, oleic acid being the main one, was inversely associated with BP; however, this is contrary to the results from earlier studies conducted mainly in the United States of America and Northern Europe [73]. This may be due to the difference in food sources of MUFA: dairy, nut butter, and meat for the Western diet, and olives and olive oil for the traditional Mediterranean diet [74,75].

SCFAs, mainly acetate, propionate, and butyrate, result from fermentation from different bacteria and production pathways [76]. In the studies presented in Table 1, particularly in Calderón-Pérez et al. [47], it appears to be a decrease in SCFAs in patients with hypertension. There is indeed accumulating evidence that SCFAs levels and relative gut bacteria have a lower abundance in patients with hypertension than in those without. Consistently, an increase in SCFAs levels can have a protective effect on hypertension [76]. The effects of dieting with SCFAs were demonstrated in various studies. One study by Roshanravan et al. showed that the supplementation with butyrate and insulin results in a decrease in systolic and diastolic pressure in patients with diabetes mellitus [77]. A different study by Marques et al. showed that dietary supplementation with fiber results in increasing acetate and the downregulation of the renin–angiotensin system (RAS), reduction in BP, and improvement of cardiovascular health and function [78].

### 2.3. Inflammation

Inflammation has several beneficial effects, such as eradicating pathogens and protecting organs from damage, but when its regulation is imbalanced it can lead to severe and prolonged inflammatory responses. These, in turn, may result in progressive tissue damage, organ dysfunction, fibrosis, and potentially, systemic inflammatory response syndrome. Overwhelming evidence shows that low-grade chronic inflammation contributes to the initiation and maintenance of essential hypertension due to the modulation of both innate and adaptive responses [79,80,81]. Several factors may contribute to the inflammation, particularly higher levels of phosphatidylcholines (PCs) and LysoPCs, as well as sphingomyelins and ceramide. Indeed, as observed in Table 1, several studies have shown an increase in these metabolites [32,34,41,48,51]; however, a recent study shows a decrease in a LysoPC [35].

LysoPCs result from the conversion of PCs by a pro-inflammatory mediator, phospholipase A2 (PLA2) [82]. LysoPCs have many biological functions in organisms, such as pro-inflammatory, oxidative stress, apoptosis induction, and anti-infective effect, being implicated in various inflammatory diseases, such as atherosclerosis by altering different physiological functions in different cell types [83,84]. Indeed, lysoPCs trigger various downstream signaling pathways, such as MAPK and NF-κB, via G protein-coupled receptors and Toll-like receptors, mediates numerous biological functions, like chemotaxis induction, inflammatory factors release, oxidative stress, and apoptosis modulation [83]. There is evidence that lysoPC induces the uncoupling of eNOS, which contributes to vascular relaxation and integrity, and reactive oxygen species (ROS) production [85,86], blocks NO bioactivity [87] and decreases NO production via ERK1/2 activity [88], impairing vascular function, which might be relevant in hypertension pathogenesis. Yet, other studies have shown that lysoPC elevates eNOS expression through the JUK and ERK1/2 signaling pathways, while not affecting the p38 MAPK signaling pathway. Additionally, it enhances SP1 and AP1 transcription factors, promoting gene expression [89]. Nonetheless, researchers have uncovered that the overexpression of NO induced by lysoPC leads to oxidative stress and cellular damage in endothelial cells [83,90].

Finally, sphingolipid metabolism also appears to play a key role in the regulation of inflammatory signaling pathways [91]. Two of the most abundant sphingolipids in circulation are sphingomyelins and ceramide [92], which seem to be increased in hypertension. Sphingomyelin is hydrolyzed to phosphocholine and ceramide by sphingomyelinases, typically propagating inflammatory signaling, by the ceramide produced, either by this process of sphingomyelin hydrolysis or de novo pathway [93]. As mentioned before, ceramide mediates vascular dysfunction by inhibiting eNOS-serine/threonine protein kinases heat shock protein 90 signaling complex and inducing endothelium-dependent contraction by triggering the release of thromboxane A2 [32]. However, the roles of endogenous sphingomyelins and ceramide in inflammation are complex as the ceramide effect on inflammatory signaling, either potentiate or attenuate, seems to be dependent on the stimulus of ceramide production and the ceramide species generated [93].

### 2.4. Oxidative Stress

A strong association between oxidative stress and hypertension has already been established by several studies [94,95,96]. It has been shown that oxidative stress has multiple effects on this multiple factorial disease, resulting in vascular endothelial dysfunction via NO depletion, activation of the vascular RAS, production of angiotensin II, proliferation of vascular smooth muscle cells, and generation of ROS [97]. These mechanisms subsequently lead to vascular constriction, elevated BP, accelerated arteriosclerosis, and the development of hypertension [96,97].

Several metabolites can promote or reduce oxidative stress, and, in Table 1, it can be observed that few were altered in hypertension, such as lysoPCs and glycine, but also fatty acids and their products, like acylcarnitines. The association of amino acid metabolism with oxidative stress in hypertension has also been reported. Glycine (mentioned in Section 2.1) appears to reduce oxidative stress by contributing to glutathione synthesis, which helps reduce excess aldehydes and free radicals, promoting NO production, and participating in the synthesis of elastin and collagen [40].

As mentioned above in Section 2.3, lysoPC causes the overexpression of NO, leading to oxidative stress and cellular damage in endothelial cells, which contributes to vascular relaxation and integrity and ROS production [85,86], blockage of NO bioactivity [87], and a decrease in NO production via ERK1/2 activity [88].

Furthermore, intracellular fatty acids can have a dual effect on mitochondrial ROS production [98,99]. Firstly, it can promote superoxide generation by inhibiting the rate of electron flow through complexes I and III and complexes III and IV in the forward mode of electron transport [100,101,102,103]; on the other hand, it can lead to a decrease in superoxide production by acting as uncouplers due to their protonophoric action [100]. Thus, it remains unclear whether the buildup of fatty acids can directly trigger mitochondrial ROS generation and induce oxidative stress. Nevertheless, elevated concentrations of fatty acids might diminish the protein’s ability to bind with other metabolites, which could potentially overwhelm fatty acid-binding proteins, leaving more active metabolites, like acyl-CoAs and acylcarnitines, unbound, thus contributing to oxidative stress [104].

In the future, some of the metabolites identified in Table 1 could be used as biomarkers of hypertension. However, for a metabolite to be considered a biomarker, several validations must first be carried out and it must fulfil some specific requirements.

Table 2 presents a summary of the data discussed throughout this article according to amino acid metabolism, fatty acid metabolism, inflammation and oxidative stress. It was designed to provide a clear and organized overview of the most relevant information, facilitating the understanding of the key points addressed.

**Figure 2:**
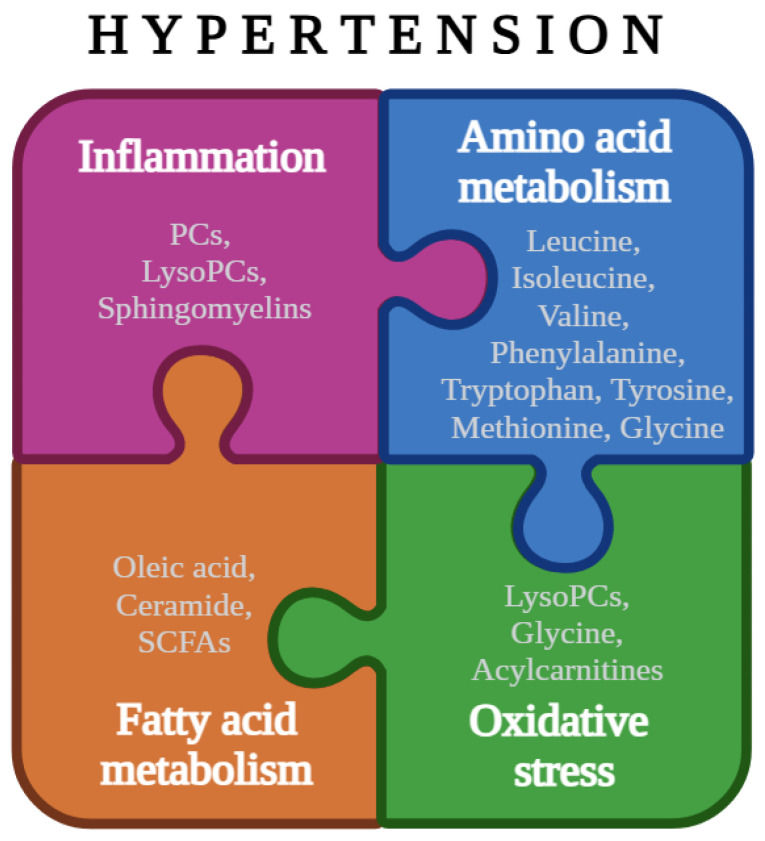
Overview of the interactions between the metabolic pathways discussed, highlighting their connections and supporting the understanding of the data (created with Biorender).

Figure 2 shows the interactions between the metabolic pathways involved, illustrating the connections between the pathways discussed and potentially aiding in the understanding of the information.

## 3. Limitations and Future Perspectives

Metabolomics has emerged as a powerful tool for biomarker identification due to its ability to provide a comprehensive snapshot of metabolic dynamics in response to pathophysiological stimuli or genetic modifications. This approach is particularly valuable for understanding interactions between an organism and its environment, such as the impact of diet on cardiovascular disease [105]. The integration of metabolomics with other “omics” approaches, such as proteomics, enhances the understanding of complex biological systems and facilitates the identification of novel biomarkers [106,107].

NMR and MS are key analytical tools in metabolomics, offering structural information, and absolute quantification of metabolites without requiring exogenous standards. These features make both NMR and MS particularly advantageous for large-scale and long-term studies, such as epidemiological research [108,109]. Despite these advantages, these techniques also have some limitations, as described above in Section 2. Briefly, NMR has low analytical sensitivity, only detecting metabolites in higher abundance in the samples. While MS, on the other hand, has low reproducibility, requires more sample preparation, is a destructive method, and the identification of metabolites is complex [31]. As for FTIR spectroscopy, it is a relatively simple technique, with little processing of the sample, allowing analysis of various groups of biomolecules. However, this technique does not allow specific molecules to be identified, and peak identification is relatively hard and time-consuming [28]. Besides that, these techniques require careful optimization of experimental conditions to minimize the influence of confounding variables such as diet, stress, lifestyle, and medication use on metabolic composition [110].

Metabolomics has been extensively applied to cardiovascular disease research, providing insights into metabolic disturbances associated with conditions like heart failure and ischemic heart disease. By profiling metabolites in biofluids and tissues, researchers can identify new biomarkers and pathophysiological pathways, which may inform therapeutic targets and improve risk stratification and personalized medicine approaches [110,111]. The use of metabolomics in cardiovascular research is further complemented by proteomics, which aids in quantifying changes in metabolites and their corresponding enzymes [107].

Despite its potential, metabolomics faces several challenges, including the need for large population studies to identify robust biomarkers and the intrinsic limitations of biological and experimental measurements. The complexity of metabolic data, influenced by numerous factors such as diet and comorbidities, necessitates careful study design and sample selection to avoid confounding results. Recommendations for improving metabolomics research include optimizing sample collection, preparation, and data analysis, as well as involving clinicians in study design to enhance the clinical relevance of findings [110,111].

A biomarker is defined as a measurable characteristic that can indicate the presence or absence of disease, biological processes or responses to therapeutic interventions. There are various types of biomarkers, depending on the outcomes analyzed, including diagnostic, risk, monitoring and prognostic biomarkers [112,113,114]. For a metabolite to be considered a biomarker, several validations must first be carried out, including analytical validations, sensitivity and specificity tests and clinical validation [112]. Besides that, a biomarker should be reproducible, stable and easy to measure [115]. These challenges might be overcome through multi-omics approaches [116] that by combining multiple biological domains could provide a more comprehensive molecular understanding of hypertension and cause and effect chain, which provides essential insight for novel therapies.

Another approach is the use of Artificial Intelligence tools, particularly machine learning, which can be employed for processing metabolomics data as well as identifying molecules and analyzing pathways [117]. These tools have the capacity to uncover hidden patterns, enhancing the precision of biomarker identification. Moreover, the continuous development of more powerful information acquisition and analysis tools, along with advanced databases and software, further supports the effective processing of complex data. This combination not only accelerates biomarker discovery but also improves the accuracy and reproducibility of the results, ultimately contributing to the advancement of personalized medicine.

## 4. Conclusions

The application of metabolomics approaches, such as FTIR spectroscopy, NMR spectroscopy, and MS, has significantly advanced the understanding of hypertension. Indeed, there is an increasing interest in the appliance of metabolomics to study these diseases by using biological fluids, such as plasma and serum. The main studies are focused on NMR and MS-based approaches and show a diversity of metabolites that are altered with individuals with hypertension, showing the complexity of hypertension pathophysiology, as the metabolites identified are involved in multiple metabolic pathways, namely amino acid metabolism, fatty acid metabolism, oxidative stress, and inflammation.

Finally, these techniques enable comprehensive profiling of metabolites, providing invaluable insights into the complex biochemical alterations associated with these conditions. Collectively, these metabolomic tools enhance the ability to identify novel biomarkers, understand disease mechanisms, and develop targeted therapeutic strategies. The integration of FTIR, NMR, and MS in metabolomics underscores their relevance and potential in unraveling the complexities of CVD and hypertension, paving the way for personalized medicine and improved clinical outcomes.

## Figures and Tables

**Table 2:** The main metabolites associated with hypertension, identified in human samples using metabolomics.

Metabolic Pathway	Techniques	Metabolites	References
Amino Acid Metabolism *	MS and NMR	↑ Leucine	[45,46,48]
		↓ Isoleucine	[45,46,48]
		↓ Valine	[48,56]
		↑ Phenylalanine	[40,46,48]
		↓ Tryptophan	[32,48,56]
		↓ Tyrosine	[48,54,56]
		↑ Methionine	[32,48,53,56]
		↓ Glycine	[8,40,45,46,52,54,56]
Fatty Acid Metabolism	MS and NMR	↑ Oleic acid	[15,32,51]
		↑ Ceramide	[32]
		↓ SCFAs	[44,46,47]
Inflammation	MS, NMR and FTIR	↑ PCs	[36,48,49,50,51]
		↑ LysoPCs	[32,35,48,51]
		↑ Sphingomyelins	[46,48]
Oxidative stress	MS and NMR	Acylcarnitines	[48]

↑, increased levels; ↓, decreased levels. Abbreviations: FTIR, Fourier transform infrared spectroscopy; MS, mass spectrometry; NMR, nuclear magnetic resonance; PC, phosphatidylcholine; SCFA, short-chain fatty acid. * Some contradictory information on variation amino acids were found.

## Abbreviations

The following abbreviations are used in this manuscript:

AAA	Aromatic Amino Acids
ATR	Attenuated Total Reflectance
BP	Blood Pressure
BCAA	Branched-Chain Amino Acids
CVD	Cardiovascular Disease
DBP	Diastolic Blood Pressure
FTIR	Fourier Transform Infrared
GC	Gas Chromatography
LC	Liquid Chromatography
LysoPC	Lysophoshatidylcholine
MS	Mass Spectrometry
MUFA	Monounsaturated Fatty Acids
NMR	Nuclear Magnetic Resonance
NO	Nitric Oxide
PC	Phosphatidylcholine
RAS	Renin–Angiotensin System
ROS	Reactive Oxygen Species
SBP	Systolic Blood Pressure
SCFA	Short-Chain Fatty Acids
